# Antimicrobial Resistance and Treatment in Companion, Food and Exotic Animals

**DOI:** 10.3390/antibiotics11101360

**Published:** 2022-10-05

**Authors:** Nikola Puvača

**Affiliations:** Department of Engineering Management in Biotechnology, Faculty of Economics and Engineering Management in Novi Sad, University Business Academy in Novi Sad, Cvećarska 2, 21000 Novi Sad, Serbia; nikola.puvaca@fimek.edu.rs

Antimicrobial resistance (AMR) occurs when bacteria, viruses, fungi, and parasites change over time and cease to respond to applied antibiotics, making infections more difficult to treat and increasing the risk of disease spread, severe illness, and death. The resistance to antibiotics and other antimicrobials leads to a decline in their effectiveness and a subsequent increase in the difficulty of treating infections. Antimicrobials—including antibiotics, antivirals, antifungals, and antiparasitics—are medicines used to prevent and treat infections in humans, animals, and plants [1].

AMR is a global health and development threat. To achieve sustainable development goals, multisectoral action is urgently needed. Drug-resistant pathogens are primarily caused by the misuse and overuse of antimicrobials [2]. A lack of clean water and sanitation, along with inadequate infection prevention and control, also contribute to the spread of microbes, some of which are resistant to antibiotic treatment, especially in pets and food animals. Moreover, in terms of economic costs, AMR is significant [3]. Furthermore, prolonged illness leads to longer hospital stays, more expensive medicines, and daily financial challenges for those affected. With the absence of effective antimicrobials, it will be more difficult, if not impossible, for modern veterinary or human medicine to effectively treat infections [1], including those caused by major surgery or chemotherapy.

AMR continues to threaten the ability of medical workers to treat common infections, both in veterinary and human practices, due to the emergence and spread of drug-resistant pathogens [4]. Especially alarming is the rapid global spread of bacteria resistant to existing antimicrobial medicines, such as antibiotics, which cause infections that cannot be treated.

Several groups of authors have presented review and research articles on the possible use of natural alternatives in the production and treatment of infectious diseases in food animals, such as poultry and cattle. Khan et al. [5] have pointed out that because of developing bacterial resistance and increasing public awareness of health and food safety, the use of antibiotics as growth promoters in the chicken industry has been outlawed. According to the authors of the published paper in this Special Issue (SI), the problem with AMR has spurred the poultry industry and sector to explore safe antibiotic alternatives and to focus on developing better long-term feed management solutions to improve chicken health and growth. Recently, researchers have been focusing much attention on the drumstick tree (*Moringa oleifera*), which is a natural product with many health benefits for poultry. *M. oleifera* is known for its antimicrobial, antioxidant, anti-inflammatory, and hypocholesterolemic properties. Due to the presence of hundreds of essential ingredients, *M. oleifera* can also activate digestive enzymes in the stomach. Keeping in mind the potential benefits of *M. oleifera* on poultry, Khan et al. [5] have emphasized its significant number of positive effects in their review.

Furthermore, following the European Union’s restriction on antibiotic growth promoters, research on enhancing gut health has been accelerated [6]. With the poultry industry facing issues previously controlled by antimicrobial growth promoters, the search for suitable alternatives has continued. In their next review, Khan et al. [6] describe how the use of fennel seeds (*Foeniculum vulgare*) could be beneficial for poultry. The physicochemical and biological properties of *F. vulgare* are discussed, as well as the diverse chemical composition of the plant. According to Khan et al. [6], *F. vulgare* seeds have various biological effects in poultry, such as improved performance, higher immune cell proliferation, reduced oxidative stress, and boosted antibody titers against infectious diseases. The published review in this SI focuses on the effects of *F. vulgare* seeds as a feed additive in poultry production as a natural alternative to antibiotics.

On the other hand, in the Republic of Serbia, Kovačević et al. [7] have investigated a new perspective on origanum (*Origanum vulgare* L.) and winter savory (*Satureja montana* L.) essential oils as bovine mastitis treatment alternatives. Kovačević et al. [7] researched strains derived from aseptic milk samples collected from Holstein–Friesian cows with clinical or subclinical mastitis during morning milking. Clinical mastitis was assessed by clinical examination, while subclinical mastitis was confirmed using somatic cell count in the milk samples. The tested essential oils have shown promising antimicrobial activity and could be considered one of the treatment approaches for mastitis-affected cows, according to results obtained at the end of the research [7].

When it comes to companion animals, research conducted in Romania by Dégi et al. [8] has focused on antimicrobial drug-resistant *Salmonella* in urban cats and raised the question of whether there is an actual risk to public health. The authors investigated the presence of *Salmonella* spp. in the feces of client-owned cats in urban areas and evaluated the risk posed to public health. All collected samples were individually screened for *Salmonella* spp., following molecular testing for the presence of the *invA* gene in all of the *Salmonella* spp. isolates. The authors found that all of the tested strains showed strong resistance toward cefazolin, cefepime, ceftazidime, and ceftriaxone [8]. Additionally, resistance was observed to trimethoprim/sulfamethoxazole, ampicillin, ampicillin/sulbactam, gentamicin, nitrofurantoin, and amikacin. The results of these studies showed that substantial public health issues and medical concerns, especially for vulnerable people, such as children, the elderly, and immunocompromised individuals, are present [8].

Research on exotic animals, such as the African fat-tailed gecko (*Hemitheconyx caudicinctus*), was conducted by Hyeon et al. [9] to investigate the genomic features of *Salmonella enterica* subspecies houtenae Serotype 45:g,z51:- in the United States. *Salmonella enterica* subsp. *houtenae* (*S. houtenae*) is a common subspecies in reptiles and has been implicated as a source of serious and life-threatening diseases in humans. Although the incidence of *S. houtenae* infections has been extensively studied, the genetic characteristics of *S. houtenae* remain largely unknown because high-quality genome sequences are missing. In their investigation, Hyeon et al. [9] obtained the complete genome sequence of *S. houtenae* 45:g,z51:- strain 20-369 isolated from multiple abdominal abscesses of *H. caudicinctus* using Nanopore and Illumina sequencing technologies and generated the 4.65 Mbp complete genome sequence of the *S. houtenae* strain 20-369. This study provides the basis for understanding the possible genetic mechanism underlying the pathogenicity of *S. houtenae* 45:g,z51:- as well as a high-quality genome reference for future comparison studies.

Furthermore, research was focused on investigating the incidence and molecular characterization of extended-spectrum β-lactamase producing *Enterobacterales* (ESBL-PE) in dogs [10], with the findings that healthy dogs may be colonized with ESBL-PE multidrug-resistant (MDR) strains, as well as humans, which may serve as a possible source for AMR. Additionally, contemporary perspectives for fluoroquinolone therapy in canine pyoderma and predisposition and resistance mechanisms in skin pathogens were investigated [11], with findings of fluoroquinolone resistance associated with methicillin-resistant staphylococci. Therefore, Azzariti et al. [11] recommended that fluoroquinolone use should be prudently guided by susceptibility testing. Debergh et al. [12] have reported the very first finding of MDR *Klebsiella pneumoniae* of sequence type 11 carrying *bla_SCO-1_* and *bla_DHA-1_* isolated from a four-month-old dog in Belgium. More importantly, this first report of the *bla_SCO-1_* gene on a conjugative IncFIB(K) plasmid is worrying as it can increase the risk of transmission to humans, animals, and the environment [12].

This Special Issue is the second volume of the previous Special Issue on “Optimization of Veterinary Antimicrobial Treatment in Companion and Food Animals” [13] and has welcomed papers focused on the latest knowledge and innovations in AMR and the optimization of veterinary antimicrobial and natural alternatives to antibiotics use in pets and exotic and food animals.
